# Modified posterior osteotomy for osteoporotic vertebral collapse with neurological dysfunction in thoracolumbar spine: a preliminary study

**DOI:** 10.1186/s13018-023-04189-3

**Published:** 2023-09-15

**Authors:** Zhisheng Long, Feipeng Gong, Long Xiong, Jiabin Wen, Gang Chen

**Affiliations:** 1 Medical College, Nanchang University, Nanchang, 330006 Jiangxi China; 2 Department of Orthopedics, Jiangxi Provincial People’s Hospital, The First Affiliated Hospital of Nanchang Medical College, Nanchang, 330006 Jiangxi China; 3 Department of Orthopedics, The Second Affiliated Hospital of Nanchang University, Nanchang, 330006 Jiangxi China

**Keywords:** Osteoporosis vertebral collapse fracture, Neurological dysfunction, Surgical treatment, Osteotomy, Kyphosis

## Abstract

**Objective:**

The risk of osteoporotic vertebral collapse (OVC) associated with delayed neurological dysfunction (DND) is substantial, and performing surgery for this condition in elderly patients presents challenges. The focus of the current research is on simplifying surgical procedures while maintaining their effectiveness. This study was designed to contribute clinical data supporting the use of modified posterior osteotomy for treating thoracolumbar OVC with DND. The study compares perioperative clinical parameters, imaging data characteristics, and changes in efficacy outcome indicators to provide evidence for the advancement of this technique.

**Methods:**

A total of 12 patients diagnosed with osteoporotic vertebral collapse and neurological dysfunction were included in the study. All patients underwent modified posterior osteotomy. Data regarding perioperative and radiological parameters as well as complications such as surgery duration, blood loss, ASIA grade, VAS, ODI, regional kyphosis angle (RKA), anterior vertebral height ratio (AVHr), and spinal canal clearance ratio (SCCr), were collected retrospectively. These parameters were then analysed to evaluate the clinical efficacy and safety of the modified posterior osteotomy technique.

**Results:**

A total of 12 patients were included in the study, with a mean age of 65.5 ± 9.7 years. The average follow-up period was 29.4 ± 5.0 months. The mean operative blood loss was 483.3 ± 142.0 ml, and the average operative time was 3.7 ± 0.7 h. The visual analogue scale (VAS) score decreased from a preoperative value of 5.8 ± 0.7 to a final follow-up value of 1.3 ± 0.8 (*P *< 0.05), indicating a significant improvement in pain. The ODI decreased from 65.2 ± 6.0 before surgery to 20.5 ± 7.0, indicating a decrease in disability, and the postoperative neurological function showed a significant improvement. Correction of the RKA was observed, with the angle changing from 35.8 ± 10.8° before surgery to 20.0 ± 3.5° after surgery and to 22.5 ± 3.1° at the final follow-up. Similarly, correction of the AVHr was observed, with the height changing from 39.3 ± 18.0 to 63.0 ± 14.3 after surgery and to 53.9 ± 8.9 at the final follow-up. Correction of the SCCr was also observed, with the ratio changing from 54.9 ± 5.4 to 68.1 ± 5.3 after surgery and to 68.68 ± 6.76 at the final follow-up.

**Conclusions:**

Posterior modified osteotomy is an effective treatment for thoracolumbar osteoporotic fractures with OVC combined with DND. It can significantly preserve vertebral height, increase vertebral canal volume, correct kyphotic angle, and improve postoperative neurological function. The simplified osteotomy also offers advantages in terms of operating time, blood loss, postoperative VAS score, and improvement in lumbar function.

## Introduction

As the global population ages, there is a growing prevalence of osteoporotic fractures. According to the current literature, approximately 54 million Americans aged 50 and above are diagnosed with osteoporosis, with 2 million experiencing osteoporotic vertebral compression fractures (OVCFs). These fractures predominantly occur in the thoracolumbar region, specifically at T11, T12, L1, and L2 [1].

For osteoporotic vertebral compression fractures, conservative treatment can lead to bone healing, reduced pain, and mild kyphosis. However, if conservative treatment is unsuccessful, progressive osteoporotic vertebral collapse (OVC) may result [2]. Taneichi et al. reported that 13.5% of patients with OVCF experience pseudarthrosis, and approximately 3% of OVC patients may experience secondary nerve damage due to displacement of bone blocks and spinal stenosis [3]. Osteoporosis itself can hinder the healing of vertebral fractures, while the progressive loss of vertebral height and increasing kyphosis accelerate the onset of spinal instability. The two main factors that substantially impact patients' quality of life are the progressive kyphosis deformity that develops after vertebral collapse and spinal cord compression caused by fracture fragments that fail to heal and move after the fracture, accompanied by varying degrees of neurological dysfunction [4, 5]. Surgical intervention is recommended for patients experiencing severe symptoms, such as severe pain, deformity, and neurological symptoms. The surgeon will determine the appropriate surgical method based on factors such as the degree of vertebral collapse and kyphosis, presence of pseudarthrosis, patient age, and neurological symptoms [6]. Some common surgical methods include vertebroplasty, posterior decompression and fusion, anterior reconstruction after spinal resection, and osteotomy. Vertebroplasty is a relatively simple and noninvasive procedure, but its effectiveness in relieving nerve compression in patients with severe OVCF and nerve injury is uncertain [7]. Traditional surgical methods for treating osteoporotic vertebral collapse (OVC) and delayed neurological deficit (DND) include anterior approach surgery, posterior approach surgery, and anterior posterior approach surgery. Posterior approach surgery can be further divided into posterior decompression and posterior spinal reconstruction, posterior spinal shortening osteotomy with direct nerve decompression, posterior indirect nerve decompression, and short segment spinal fusion combined with vertebroplasty. However, there is still some controversy regarding the optimal surgical procedure for OVC and DND. It is known that osteoporotic fractures with vertebral collapse often occur in the thoracolumbar region, with the fracture lesion located in the anterior part of the spine. Anterior surgery allows for direct treatment of the affected bone mass and decompression of the compressed area of the spinal cord. However, it has limitations in correcting kyphosis and can result in major surgical trauma [8]. Traditional posterior surgery involves posterior osteotomy, internal fixation, and local spinal decompression. This approach is familiar to spine surgeons and can effectively correct kyphosis by removing posterior elements such as the vertebral lamina and adjacent ligaments. The combined anterior and posterior approach offers advantages in terms of orthopaedic efficiency, spinal cord decompression, internal fixation loosening, and vertebral height loss. However, it is associated with considerable trauma, long operative times, and high technical requirements [9]. For patients with OVCF and neurological dysfunction, there are currently various attempts to improve surgical methods, such as modified PSO osteotomy [10], PVO osteotomy [11], transpedicular cage implantation correction, asymmetric osteotomy correction decompression, and internal fixation [12].

Our team has developed a simplified posterior osteotomy method that involves removing a portion of the vertebral lamina, the small articular process, and part of the posterior wall of the vertebral body at the affected site. Special instruments are then used to press the vertebral body from front to back, achieving decompression, lifting of the vertebral body, and moderate correction of kyphosis. In our preliminary study, we observed accurate therapeutic effects and a simplified surgical procedure, resulting in a significantly shorter operation time, reduced bleeding, and decreased operation-related complications.

## Materials and methods

All patients admitted to the Jiangxi Provincial People's Hospital from October 2016 to October 2020, diagnosed with osteoporosis and fractures, had varying degrees of neurological dysfunction on admission. This study was approved by the Ethics Committee of the Jiangxi Provincial People's Hospital. All patients included in the study underwent modified osteotomy surgery and were followed up for more than 24 months postoperatively. All patients underwent routine anterior and lateral spinal X-rays, thoracic and lumbar MRI, and thoracic and lumbar CT, and bone density was assessed before surgery using dual-energy X-ray absorptiometry (DEXA) T scores and bone turnover markers.

The patient inclusion criteria were as follows: (i) the patient is diagnosed with osteoporotic fracture according to imaging classification, accompanied by varying degrees of vertebral collapse, and the shadow phenomenon can be seen with protruding bone fragments in the vertebral canal; (ii) c treatment is ineffective; (iii) neurological impairment: below Frankel E level; (iv) bone density test standard: osteoporosis, *T* value ≤ 2. 5; (v) retrospective study with complete patient data; (vi) fractured vertebral body located in the thoracolumbar segment.

The exclusion criteria were as follows: (i) patients with severe underlying systemic diseases who could not tolerate surgery; (ii) patients with vertebral collapse caused by OVCF disease with continuous multisegmental vertebral bodies; (iii) spinal metastases: pathological fractures caused by primary and secondary tumours; (iv) patients with no neurological dysfunction; (v) patients who refused to participate in the research project; and (vi) patients with acute explosive fractures caused by severe trauma.

### Operation methods

**Fig. 1:**
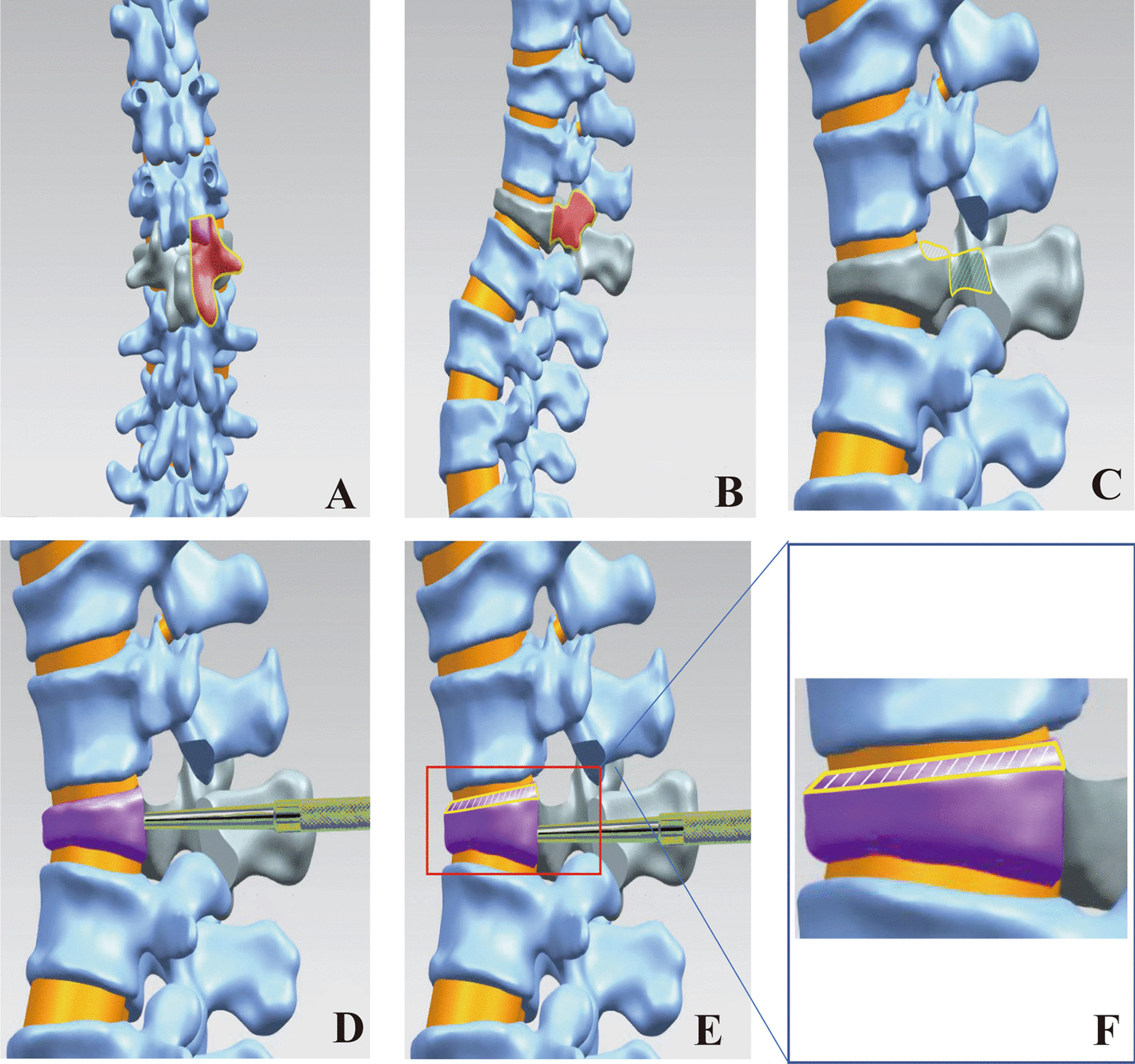
Flowchart of modified osteotomy model. **A** and **B** show the osteotomy scope, which includes one lamina, transverse process, inferior articular process of upper vertebral body, and unilateral pedicle of osteotomy vertebral body corresponding to collapsed vertebral body. **C** Excision of vertebral bone mass protruding into spinal canal. **D** Drive from the rear to the front column through a special screwdriver. **E** and **F** The anterior column and middle column of vertebral body were partially lifted, and the compression in spinal canal and kyphosis was obviously improved

Taking T12 vertebral fracture as an example, a conventional midline approach was used. Pedicle screws were inserted at 1 or 2 upper and lower vertebrae. One side of the T12 half lamina, T11 part of the lower articular process, and T12 unilateral pedicle were resected with a bone knife (Fig. 1A and B). The ligamentum flavum was removed; the spinal canal was entered from one side to expose the dural sac and entered from the back to expose the posterior wall of the vertebral body. The bleeding was thoroughly stopped, the posterior wall of the vertebral body was freed, bone blocks that markedly protruded into the vertebral canal were removed, and care was taken to protect the nerves during surgery. If there was an outstanding gap between the dural sac and the posterior wall of the vertebral body, a specially designed L-shaped screwdriver was placed on the surface of the vertebral body (Fig. 1C, D). During surgery, an L-shaped screwdriver was carefully inserted between the dural sac and the protruding bone; due to osteoporosis and nonunion of the fracture, the bone mass entering the spinal canal would begin to move away from the spinal canal to achieve decompression and partial elevation of the vertebral body. In the same way, the protruding bone mass close to the midline and on the opposite side was moved away from the spinal canal (Fig. 1D, E). Bone grafting was performed with autolocal bone and allogeneic bone on the posterolateral side. Four pedicle screws were implanted in each of the upper and lower vertebrae of the affected vertebra, and prebent titanium rods were used to connect the ends of the screws. The kyphosis deformity was appropriately corrected, and if necessary, the screw trajectory was reinforced with bone cement (Fig. 4).

### Imaging analysis

**Fig. 2:**
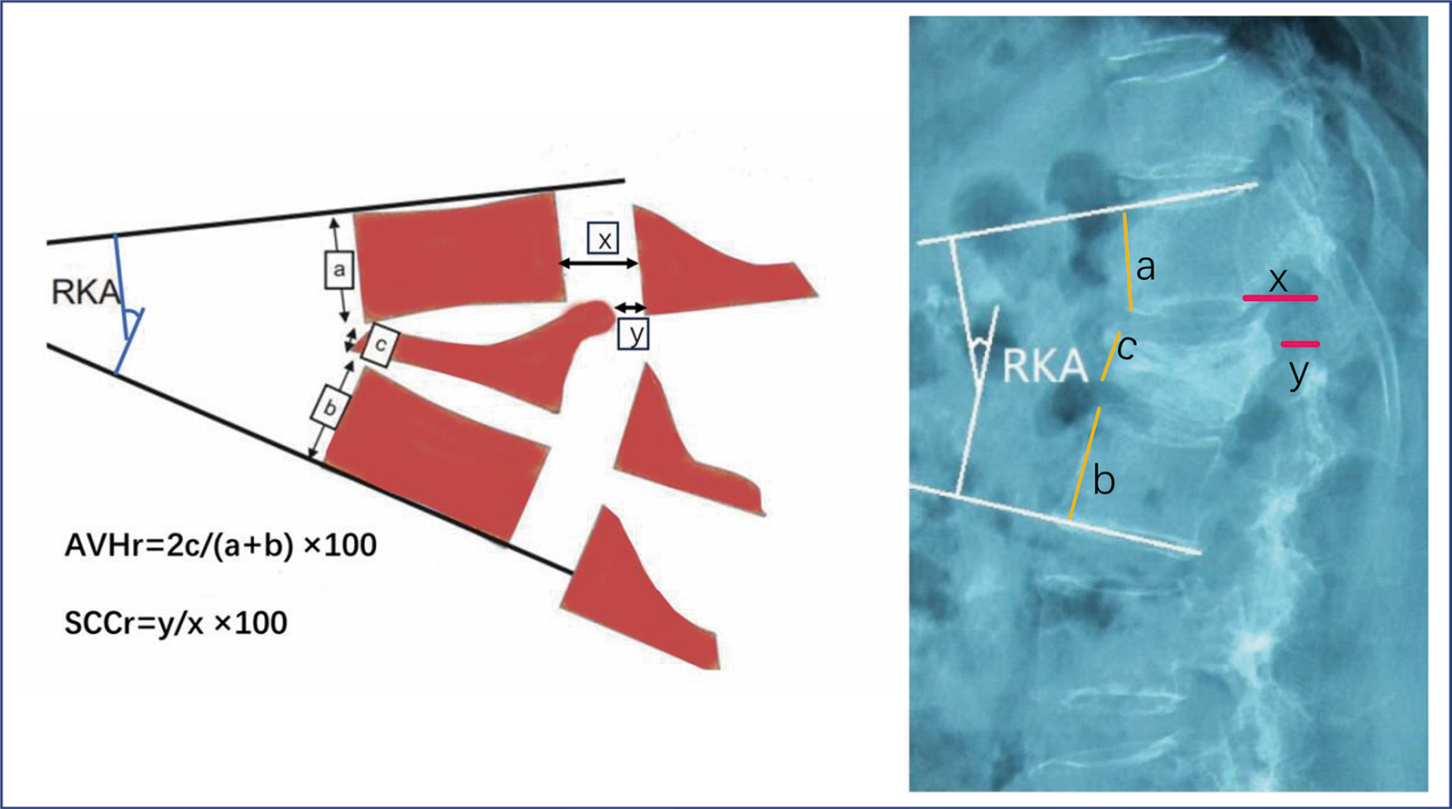
Imaging measurement [12]. AVHr is defined as the ratio of the anterior vertebral height to the adjacent unfractured anterior vertebral height. The specific calculation method is as follows: the anterior edge height a of the fractured upper vertebral body, the anterior edge height b of the fractured lower vertebral body, the anterior edge height c of the fractured vertebral body, AVHr = 2 * *C*/*a *+ *b* * 100. RKA is defined as the angle between the lower endplate of the fractured vertebral body and the lower endplate directly above the vertebral body. SCCr is the spinal canal clearance ratio, calculated as the ratio of the diameter of the vertebral canal at the compression site (y) on the sagittal X-ray to the diameter of the upper vertebral canal (x), SCCr = *y*/*x* * 100

Imaging analysis included assessment of the anterior vertebral height ratio (AVHr), regional kyphotic angle (RKA), which is used to assess local deformities, and spinal canal clearance ratio (SCCr) during preoperative, postoperative, and final follow-up. The specific calculation method is shown in Fig. 2 [12]. Imaging parameters were measured twice by a spinal surgeon with 13 years of experience, and the average values were recorded. All imaging data were measured by Digimizer 5.7.5 (MedCalc Software, Ltd, Belgium).

### Clinical outcomes

Back pain was scored preoperatively and at each routine postoperative clinical follow-up (at 1 week and at final follow-up) on a visual analogue scale (VAS, from 0 to 10, 0: no pain, 10: maximum pain) and the Oswestry Disability Index (ODI, from 0 to 50, with higher scores indicating severe disability). Neurological status was evaluated by the ASIA grading system. Operative time, blood loss, and complications related to anaesthesia and surgical procedures were also recorded.

### Statistical analysis

IBM SPSS statistical software v24.0 was used for analysis. Single-factor analysis of variance (ANOVA) was used to meet homogeneity of variance, and LSD t test was used for pairwise comparisons between groups; if homogeneity of variance was not met, Welch's analysis was used, and Dunnett's T3 test was used for pairwise comparisons between groups; a two-sample paired t test was used for pre- and postoperative VAS scores and ODI changes. All results with *P *< 0.05 were considered statistically significant.

## Results

### Demographics data

Twelve patients were included in this study, including 2 males and 10 females, with a mean age of 65.0 ± 9.7 years. The lesion segment of vertebral collapse was T11:T12:L1 at 2:9:1, with a BMD of − 3.7 ± 0.9. The mean follow-up was 29.4 ± 5.0 months, with a mean operative blood volume of 483.3 ± 142.0 ml and a mean operative time of 3.7 ± 0.7 h. The specific values are detailed in Tables 1 and 2.

**Table 1:** Demographics data

No	Sex	Age	Collapsed vertebrae	Surgical segment	BMD	ODI Pre-op	ODI Post-op	VAS Pre-op	VAS Post-op	NF Pre-op	NF Post-op	Follow-up (month)	Blood (ml)	Operation time (hour)
1	Female	72	T12	T10-L2	− 5.7	75	25	6	2	D	E	26	450	4
2	Female	48	T12	T10-L2	− 2.7	65	14	5	1	D	E	28	350	3
3	Female	66	T12	T8-L3	− 3.5	70	22	6	1	D	D	36	500	3
4	Female	64	T12	T9-L3	− 3.4	57	13	5	1	D	E	27	800	4
5	Female	81	T11	T9-L2	− 4	67	14	6	2	D	E	32	300	4
6	Female	68	L1	T11-L3	− 3	62	27	5	0	D	D	28	650	4
7	Female	77	T11	T9-L2	− 3.2	61	25	6	2	D	E	32	500	4.5
8	Male	60	T12	T10-L2	− 3.7	69	26	7	1	D	E	29	450	2.5
9	Male	59	T12	T10-L2	− 3.1	55	10	5	0	D	E	32	350	4
10	Female	64	T12	T9-L3	− 4.8	63	23	6	2	D	D	35	600	4.5
11	Female	74	T12	T10-L2	− 3.7	73	32	6	2	C	D	17	450	3
12	Female	53	T12	T9-L3	− 3	65	15	7	1	D	E	31	400	4
Ave	–	65.0 ± 9.7	–	–	− 3.7 ± 0.9	65.2 ± 6.0	20.5 ± 7.0	5.8 ± 0.7	1.3 ± 0.8	–	–	29.4 ± 5.0	483.3 ± 142.0	3.7 ± 0.7

*ODI* Oswestry Disability Index, *VAS* visual analogue scale, *NF* neurological function (ASIA grading system), *Pre-op* pre-operation, *Post-op* postoperation, *BMD* bone mineral density, *Ave* average

**Table 2:** Preoperation and postoperation of radiological data

No	Pre-op RKA (degree)	Post-op RKA (degree)	FU RKA (degree)	Pre-op AVHr (%)	Post-op AVHr (%)	FU AVHr (%)	Pre-op SCCr (%)	Post-op SCCr (%)	FU SCCr (%)
1	35.4	21.7	25	25.9	58	49.3	54.8	77.1	66.8
2	29.1	15	22.5	28.6	82.4	52.9	54.9	68.6	61.9
3	30.6	19.6	21.3	21.9	37.3	38.8	55.2	61.7	62.6
4	53.6	24.9	26.5	60.9	73.5	62.4	46.3	74.5	65.6
5	28.1	14.9	17.5	28.8	51.6	54.5	45.6	66.7	61.1
6	26.6	21.8	19.8	47.8	72.5	50.1	54.8	63	81.5
7	53.1	18.9	25.9	20.8	52.0	44.6	53.7	70.8	65.6
8	25.3	16.2	21.4	51.0	69.7	56.6	60.7	71.6	75.3
9	33.5	19.9	20.3	58.6	80.8	70.1	56.0	60.9	67.2
10	50.7	23.1	26.7	17.2	54.8	55	65.5	64.8	69.7
11	25.1	18.9	19.1	39.6	49.6	46.9	53.1	64.8	67.0
12	38.5	25.5	23.7	70.7	74.2	65.5	58.0	73.1	79.9

*AVHr* anterior vertebral height ratio, *RKA* regional kyphotic angle, *SCCr* spinal canal clearance ratio, *FU* final follow-up

### Clinical outcomes

#### Neurological function

**Fig. 3:**
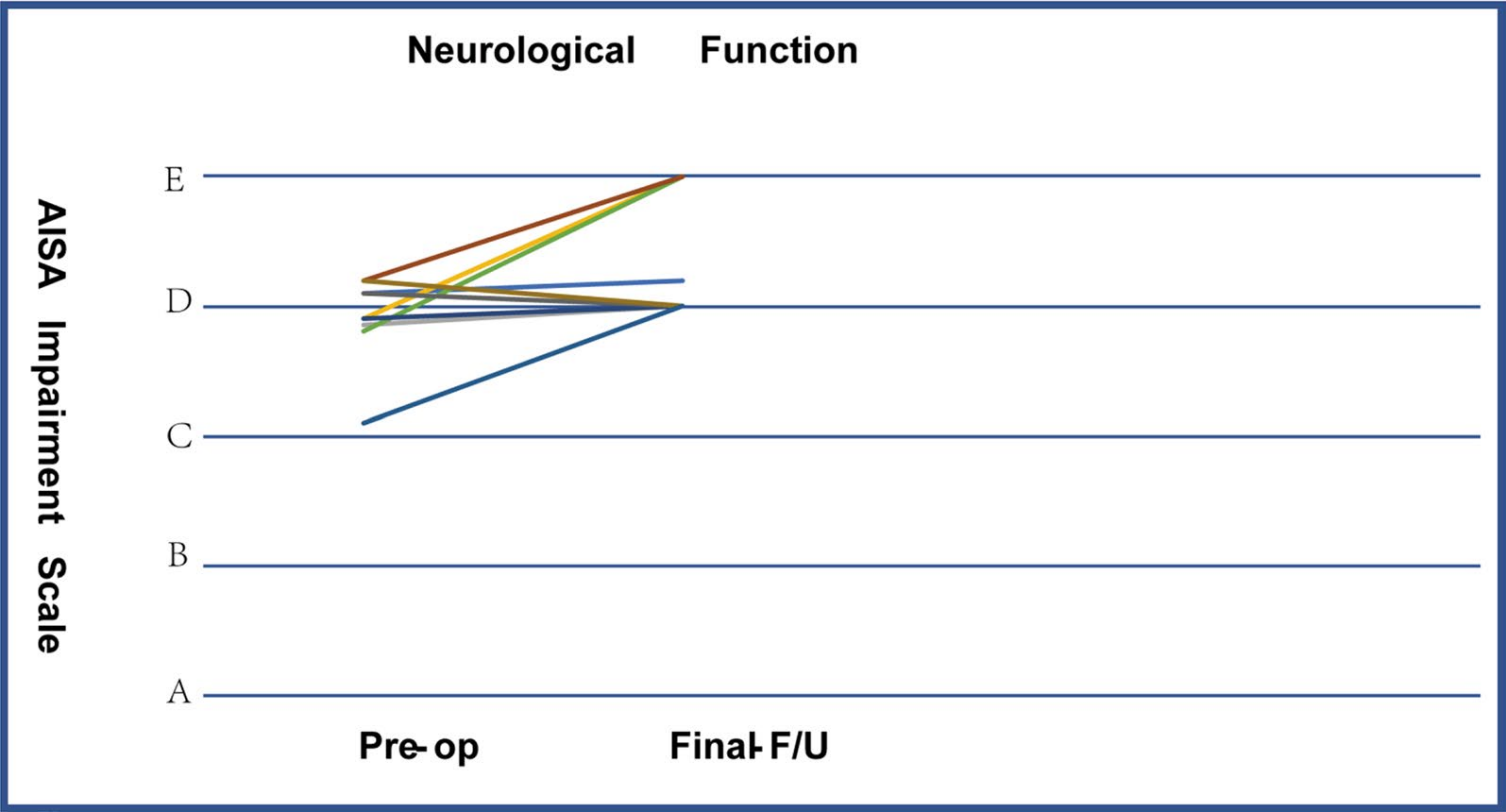
Neurological function scores of patients preoperatively and at final follow-up

According to the ASIA grading of neurological deficits, preoperatively, 1 patient was Grade C, and 11 patients were Grade D. At the final follow-up, 1 patient improved to Grade D, 7 patients improved to Grade E, and 3 patients showed no significant improvement (Grade D). Statistical analysis of preoperative and postoperative neurological function scores showed that both had statistical significance (*P *< 0.05) and that postoperative neurological function improved significantly (Fig. 3, Table 2).

#### Visual analogue scale

The VAS score for visual pain decreased from 5.8 ± 0.7 before surgery to 1.3 ± 0.8 at the final follow-up (*P *< 0.05). The VAS score at the end of follow-up was significantly reduced. Statistical analysis of both showed statistical significance (*P *< 0.05) (Tables 2 and 3).

**Table 3:** Analysis prior to and following surgery

	Preoperative	Post-op	FU
VAS	5.8 ± 0.7	–	1.3 ± 0.8
*P* value	–	–	0.000^#^
ODI	65.2 ± 6.0	–	20.5 ± 7.0
*P* value	–	–	0.000^#^
RKA	35.8 ± 10.8	20.0 ± 3.5	22.5 ± 3.1
*P* value	–	0.001^#^	0.004^#^
	–	0.229^&^	
AVHr	39.3 ± 18.0	63.0 ± 14.3	53.9 ± 8.9
*P* value	–	0.005^#^	0.066^#^
	–	0.205^&^	
SCCr	54.9 ± 5.4	68.1 ± 5.3	68.7 ± 6.8
*P* value	–	0.000^#^	0.000^#^
	–	0.820^&^	

*ODI* Oswestry Disability Index, *VAS* visual analogue scale, *RKA* regional kyphotic angle, *AVHr* anterior vertebral height ratio (%), *SCCr* spinal canal clearance ratio (%), *FU* final follow-up

^#^*p *< 0.05, compared with preoperation value

^&^*p *< 0.05, compared with final follow-up value

#### Oswestry Disability Index

The ODI decreased from 65.2 ± 6.0 before surgery to 20.5 ± 7.0 after surgery. At the end of follow-up, the lumbar ODI functional index improved significantly, and statistical analysis showed statistical significance (*P *< 0.05) (Tables 2 and 3).

#### Radiological data

*RKA* The regional kyphosis angle was corrected from 35.8 ± 10.8 before surgery to 20.0 ± 3.5 after surgery. The final follow-up regional kyphosis angle was 22.5 ± 3.1, and the immediate postoperative kyphosis correction rate was 55.9%. The final follow-up local kyphosis correction rate was 62.8%. Statistical analysis showed that the kyphosis angles at immediate and final follow-up after surgery were statistically significant compared with those before surgery (*P *< 0.05). There was no significant difference in kyphosis angles between the final follow-up and surgery (*P *> 0.05) (Tables 2 and 3).

*AVHr* The height of the collapsed vertebral body increased from 39.3 ± 18.0 before surgery to 63.0 ± 14.3 immediately after surgery. The height at the end of follow-up was 53.9 ± 8.9, and the height immediately after surgery was 160.30% of the preoperative height. Statistical analysis showed that the height of the vertebral body immediately after surgery was significantly different from that before surgery (*P *< 0.05). There was no significant difference in the angle of the vertebral body height between the end of follow-up and surgery (*P *> 0.05) (Tables 2 and 3).

*SCCr* The clearance rate of the spinal canal increased from 54.9 ± 5.4 before surgery to 68.1 ± 5.3 immediately after surgery. The clearance rate of the vertebral canal at the final follow-up was 68.7 ± 6.8. Statistical analysis showed that the vertebral canal clearance rate at immediate and final follow-up after surgery was statistically significant compared to that before surgery (*P *< 0.05). There was no significant difference in vertebral volume change between the final follow-up and surgery (*P *> 0.05) (Tables 2 and 3).

### Complications

**Fig. 4:**
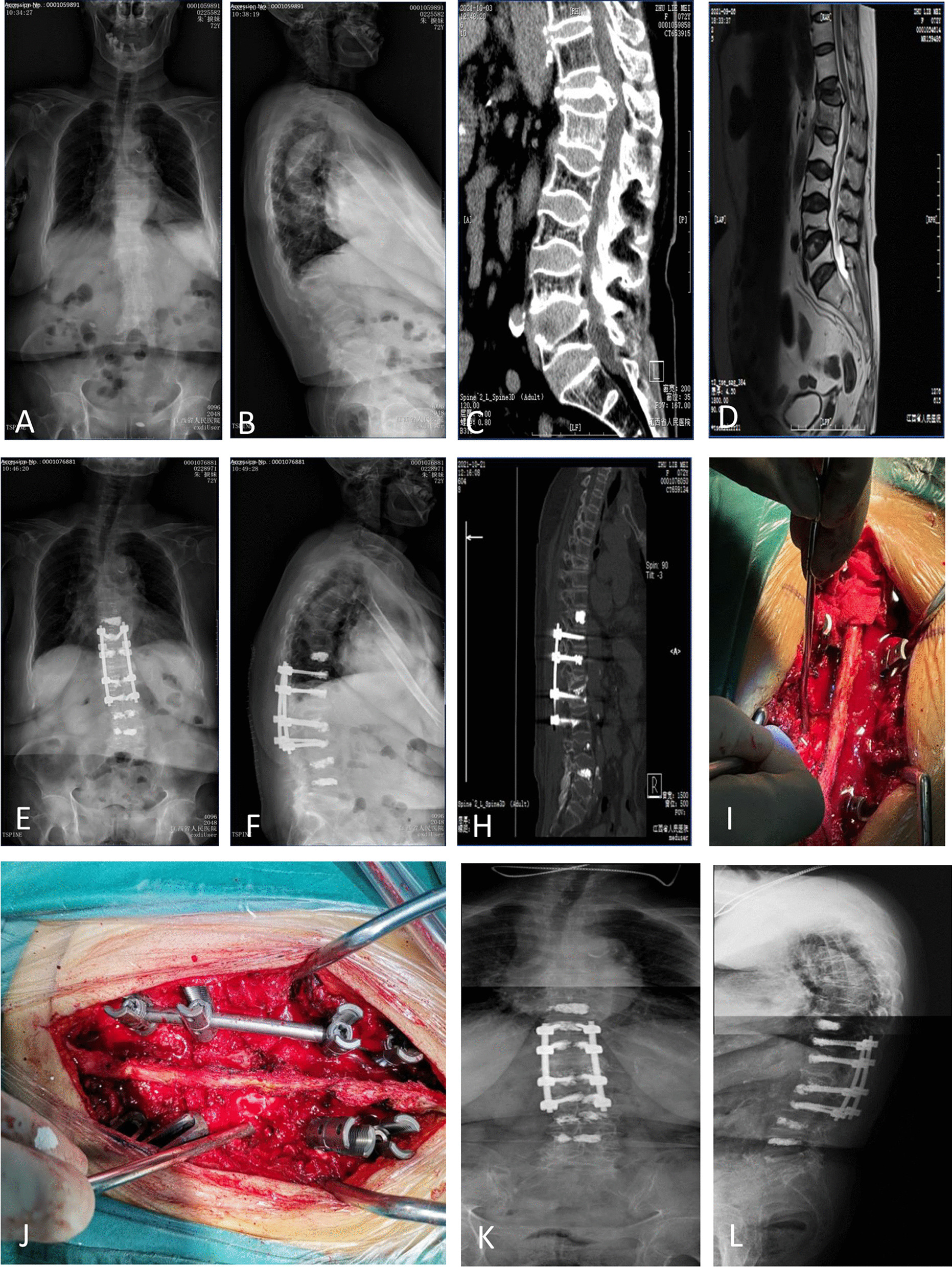
A 72-year-old female patient was admitted for low back pain and weakness in both lower limbs. Physical examination revealed local kyphosis, Franke grade D, and no pathological signs. BMD: − 5.7, VAS: 6 points, ODI: 75. T12 fracture with vertebral collapse, kyphosis deformity, RKA: 35.4°, SCCr: 54.8, AVHr: 25.9 (**A, B**). 3D CT of the spine showing a wedge-shaped collapse of the T12 with protruding bone blocks entering the spinal canal from the posterior wall (**C**). MRI of the thoracolumbar region shows significant compression of the spinal cord at T12 (**D**). Lumbar spine radiographs in both anteroposterior and lateral views. The patient underwent local laminectomy at T12 and spinal internal fixation at T10-L2, with some vertebral bodies reinforced with bone cement. Postoperative 1-week RKA: 21.7°, SCCr: 77.1, AVHr: 58.0 (**E, F**). Final follow-up, RKA: 25°, SCCr: 66.8, AVHr: 49.3 (**K, L**)

All patients had a successful operation, and there were no complications, such as wound infection, loose internal fixation, venous thrombosis, pulmonary sensation, or CSF leakage (Fig. 4).

## Discussion

The incidence of osteoporotic vertebral compression fracture (OVCF) increases with age as the population ages. Recent literature indicates that OVCFs affect more than 40% of people over 80 years old and more than 25% of postmenopausal women [13]. OVCF can lead to an increase in the kyphotic angle and localized pain, and most patients are treated conservatively. However, some patients who receive conservative treatment may experience vertebral collapse (OVC) and spinal instability, resulting in severe pain and neurological dysfunction. In such cases, surgery may be a suitable treatment option [14]. It is concerning that patients with OVCF and vertebral collapse combined with neurological dysfunction are predominantly elderly individuals with overall poor health. These patients often have multiple underlying diseases, osteoporosis, difficulties with internal fixation, and face high surgical difficulty and risk [15–17]. Currently, there are varying opinions on the definition of OVC and OVCF as well as the analysis of treatment strategies and the reasons for the occurrence of neurological dysfunction in the later stage of OVC. There is also debate regarding whether spinal decompression is necessary for the occurrence of neurological dysfunction in OVC and regarding the selection of surgical strategies for OVC combined with neurological dysfunction. The focus of research in this field is to improve treatment effectiveness, simplify treatment strategies, reduce surgical complications, and develop reasonable surgical strategies. This has become a prominent research topic [18].

### Analysing the main causes of neurological dysfunction in OVC and selecting surgical strategies without spinal decompression

The three pathological factors leading to neurological deficits in osteoporotic vertebral collapse (OVC) are nerve compression caused by posterior displacement of bone fragments in the spinal canal, progression of spinal kyphosis, and instability of the fracture site [19]. The current mainstream view suggests that neural dysfunction in OVC involves both posterior displacement of collapsed vertebral fracture blocks and kyphosis. However, there are some researchers who disagree. Ataka et al. [20] found that instability around fractures may be the primary cause of nerve damage. Malepally et al. [21] discovered through dynamic magnetic resonance imaging that the displaced bone fragments after spinal collapse have dynamic displacement and that their repeated micromovements are the main cause of spinal cord injury. Nakano et al. [22] also obtained similar results in their study, confirming that the micromovement of bone fragments and fractured segments is the main cause of SCI. The treatment goal of OVC combined with DND is primarily to restore neural function and achieve appropriate correction. According to Zhu et al. [23], the main cause of neurological dysfunction in patients with OVC combined with DND was not displaced bone blocks. The team introduced a new transpedicular bone grafting technique to address this issue. During surgery, tools such as a scraper were used to lift and elevate the vertebral body, and autologous bone particles were filled into the hollow vertebral body through the pedicle. Long-segment fixation was performed by implanting pedicle screws above and below the fracture. The researchers in this study reported that neurological function in all 24 patients who were followed up recovered to Frankle grade E. Over an average follow-up period of 38 months, significant improvements were observed in local kyphosis angle, vertebral height, VAS score, and other factors. Additionally, this technique resulted in shorter blood loss and operating time compared to traditional surgery. The study indirectly suggested that an improved osteotomy technique may have positive therapeutic effects in improving neurological function. Based on this concept, our surgical approach was modified to include the placement of pedicle screws above and below the collapsed vertebral body to achieve stabilization. Additionally, unilateral laminectomy and removal of displaced bone blocks in the anterior direction were performed, which positively impacted the recovery of neurological function. At the postoperative follow-up currently, the limited decompression and fixation of the collapsed vertebral body are found to have resulted in significantly improved neurological function.

### Traditional treatment strategy selection for OVC with DND

In a follow-up study by Nakamae et al. [24] involving 244 OVCF patients treated with PKP/PVP, including 30 cases of DND, 84% of DND patients (25 cases) experienced improvement in neurological symptoms, while 5 cases did not show a significant improvement. The current mainstream view suggests that PKP/PVP does not significantly improve the local kyphosis angle or spinal canal occupancy rate in patients. Additionally, severe fracture collapse of the vertebral body may hinder the establishment of an ideal puncture pipeline, potentially causing damage to surrounding tissues during the puncture process. Therefore, careful patient selection is necessary for this group of patients. Similar conclusions were drawn by Chang et al. [25] in their secondary classification of the literature. Consequently, when considering PVP/PKP surgery for OVC patients with DND, strict indications must be followed to avoid poor postoperative improvement of neurological dysfunction.

Traditional open surgical methods for treating osteoporotic vertebral collapse with neurological deficits include anterior decompression and direct fixation, posterior short- or long-segment surgery, and combined anterior and posterior surgery. In a study conducted by Kanayama et al. [8], the researchers emphasized the importance of anterior spinal support and reviewed 31 cases of anterior spinal reconstruction. The study found that 80% of patients achieved satisfactory results with simple anterior decompression, confirming the safety of anterior fusion surgery. Anterior surgery offers the advantage of direct visualization of the decompression site and preservation of posterior supporting tissues. Another study conducted by Sudo et al. [4] compared and analysed patients with osteoporotic thoracolumbar collapse with neurological deficits. The study compared those who underwent anterior decompression and titanium mesh support (*n *= 32) to those who underwent posterior decompression and pedicle screw fixation with vertebroplasty (*n *= 18). The results showed that the operative time was similar between the two groups, but intraoperative blood loss was significantly lower in the posterior group. This study further supports the advantages of posterior surgery in trauma. Taneichi et al. [3] reported that 50% of anterior fusion procedures require revision. In a study by Takenaka [26] on anterior surgery, 3 out of 9 patients experienced postoperative worsening of kyphosis and needed revision surgery. Therefore, when considering a multisegment anterior approach, it is important to consider the risks of surgical bleeding and collapse of the titanium mesh or implant. In comparison, the posterior approach offers a clearer anatomical structure and lower surgical difficulty. Our study further supports the advantages of posterior surgery. The cases examined in this group demonstrate that posterior surgery has certain surgical advantages over traditional anterior or posterior surgery, including higher rates of kyphosis correction, shorter operation time, reduced bleeding volume, improved neurological function recovery, and lower incidence of postoperative complications.

### Research into modified surgical techniques

Patients with OVCFs and degenerative disc disease (DDD) present unique challenges in treatment due to factors such as unstable spine, reduced bone mass, and poor internal fixation [27]. Additionally, these patients often have advanced age, multiple comorbidities, and overall poor health conditions. As a result, treatment strategies for OVCF and DDD differ significantly from those for the general population. Sung-Kyuet et al. [10] proposed an improved technique for posterior spinal osteotomy (PSO) that focuses on limited kyphosis correction. They modified the traditional PSO by changing the location of the osteotomy vertex to the anterior one-third position of the vertebral body. In their follow-up cases, the local kyphosis angle (LKA) decreased from 26.2° before surgery to 8.3° after surgery. The JOA score improved from 13.2° before surgery to 21.7° after surgery, and the lumbar functional index (ODI) decreased from 40.3° before surgery to 13.6° after surgery. The bleeding volume during the surgery was 1098 ml, which is better than that of traditional PSO. This modified technique also resulted in a shorter operation time and reduced bleeding volume. Plais et al. [28] improved the operation of the PSO technique by addressing the low local vertebral bone mass and nonunion of fractures in patients with OVC vertebral collapse. The authors utilized an endplate reamer for intervertebral fusion at different angles (vertical angles of 25° and 60°) and created a triangular osteotomy area in the central column of the vertebral body during the rotation process, thereby simplifying the PSO technology. Cianfoni et al. [16] employed an 'insert type bracket screw', which is a device similar to a bone cement balloon support device. The authors inserted an expandable metal balloon into the collapsed vertebral body through pedicle puncture and placed a transpedicular screw, along with internal fixation techniques at the upper and lower levels. This approach resulted in a 74% increase in the postoperative vertebral height of the anterior column, a 150% increase in the middle column, and a 17% increase in the posterior column, with a local kyphosis correction of 8–10°. Adachi [29] performed PVO, Lee et al. [30] inserted fusion cages into collapsed vertebrae, and Dong et al. [31] combined traditional VCR with 3D-printed artificial vertebrae (3DP-AVB).

Based on previous literature and research, our team improved the compression of the bone block into the spinal canal by tapping on the protruded bone block from the central column during surgery. The central column was appropriately elevated and supported with pedicle screw fixation through the posterior approach. At the final follow-up, there were significant improvements in VAS, ODI, regional kyphotic angle, vertebral height, and nerve function as described in the results of the paper. The simplified surgical procedure in this study resulted in significant reductions in both operating time and blood loss compared to traditional open surgery. This can be attributed to the reduced trauma experienced by the patient and the effective decompression of nerves achieved through the removal of bone fragments from the posterior wall of the vertebral canal. The patient's osteoporosis contributed to the decrease in local bone mass, allowing for partial movement and lifting of the vertebral body under the pressure of an L-shaped device. Ultimately, this led to an elevation of the vertebral body height and correction of the local kyphosis angle.

However, it is important to acknowledge the limitations of this study. First, it should be noted that this study is retrospective in nature and lacks a control group. Additionally, the follow-up period in this study is relatively short, and the sample size is not considerable, which may have an impact on certain research findings. Furthermore, the evaluation of local fusion status after osteotomy was not conducted in this study although such status could have implications for clinical effectiveness. Finally, one of the shortcomings of this study was that intraoperative neurophysiologic monitoring was not used, possibly having a negative impact on the evaluation of patient safety.

## Conclusions

Posterior modified osteotomy is a highly effective treatment for thoracolumbar osteoporotic fractures with OVC combined with DND. This procedure has been shown to significantly preserve vertebral height, increase vertebral canal volume, correct kyphotic angle, and improve postoperative neurological function. Additionally, the simplified osteotomy technique offers several advantages, including reduced operating time, decreased blood loss, improved postoperative VAS score, and enhanced lumbar function.

## Data Availability

All data used or analysed during this study are included in this published article. The datasets from this study are available from the corresponding author upon reasonable request.

## Abbreviations

ODI: Oswestry Disability Index
VAS: Visual analogue scale
RKA: Regional kyphotic angle
AVHr: Anterior vertebral height ratio
SCCr: Spinal canal clearance ratio
PSO: Pedicle subtraction osteotomy
OVC: Osteoporotic vertebral collapse
PVO: Partial vertebral osteotomy
VCR: Vertebral column resection
DND: Delayed neurological deficit
